# *Gardnerella* fibrinogen-binding protein as a candidate adherence factor

**DOI:** 10.3389/fcimb.2025.1556232

**Published:** 2025-05-08

**Authors:** Aistė Bulavaitė, Justas Dapkūnas, Raminta Reškevičiūtė, Indrė Dalgėdienė, Lukas Valančauskas, Lina Baranauskienė, Milda Plečkaitytė

**Affiliations:** Institute of Biotechnology, Life Sciences Center, Vilnius University, Vilnius, Lithuania

**Keywords:** *Gardnerella*, surface protein, bacterial vaginosis, adhesin, structure modeling, MSCRAMM, fibrinogen

## Abstract

Bacterial vaginosis (BV), a form of vaginal dysbiosis, is associated with numerous adverse reproductive and obstetric outcomes. *Gardnerella* spp. are among the key bacteria identified in most BV cases. The formation of a polymicrobial *Gardnerella*-dominated biofilm on the vaginal epithelium is a characteristic diagnostic marker of BV. *Gardnerella* colonization and biofilm formation indicate a significant adhesion potential, the determinants of which remain unexplored. In this initial approach to identify *Gardnerella* adhesins, we analyzed the Cna protein located on the *G. vaginalis* ATCC 14018 cell surface as determined previously. Structure modeling of Cna (designated Grd Cna) revealed that the protein contains N2 and N3 domains with an immunoglobulin (IgG)-like fold, which shows structural homology to the corresponding domains in SdrD and UafA proteins of the microbial surface component recognizing adhesive matrix molecules (MSCRAMMs) family. A single B domain shares structural similarity with the corresponding domain of Sdr proteins. The R region is rich in PKD repeats, while the C-terminal contains a non-canonical LVNTG cell wall sorting motif. The *cna* gene was predominantly detected in *G. vaginalis* isolates but was absent in other commonly identified *Gardnerella* species isolates. The recombinant Grd Cna protein binds dose-dependently to human fibrinogen but does not interact with fibronectin or collagen types I, III, or IV. Cna-positive *G. vaginalis* cells adhered to immobilized fibrinogen; however, recombinant Cna did not inhibit this binding, suggesting that Cna may not be a major adhesin mediating *G. vaginalis* adherence to this ECM component.

## 1 Introduction

Bacterial vaginosis (BV), a form of vaginal dysbiosis, is characterized by a reduction in beneficial lactobacilli and an increase in anaerobic bacteria, primarily *Gardnerella* spp. (Muzny et al., 2019). BV has been associated with gynecological and reproductive health issues in women (Chen et al., 2021). The specific diagnostic marker of BV is the polymicrobial *Gardnerella*-dominated biofilm coating the vaginal epithelial cells (Swidsinski et al., 2013). The attributed role of Gram-positive *Gardnerella* as the initial colonizing species in BV, its displacement of lactobacilli and persistence in the vagina, as well as its ability to form a biofilm, indicate a significant adhesion potential (Patterson et al., 2010; Alves et al., 2014). The determinants that promote *Gardnerella* adherence to host tissues have not yet been explored.

Gram-positive bacteria express a variety of cell wall-anchored proteins, collectively termed adhesins, that mediate the adherence to epithelial cells, plasma proteins, and extracellular matrix (ECM) components (Foster, 2019). Adhesins are covalently attached to the cell wall by sortase enzymes, which recognize the LPXTG motif near the C-terminus of the protein (Ton-That et al., 2004). A subset of adhesins are multisubunit protein polymers known as pili. Pili have been identified in many Gram-positive bacteria, where they mediate bacterial adherence to host cells and contribute to biofilm formation (Danne and Dramsi, 2012). The most prevalent and the best-studied group of non-pilus adhesins is the microbial surface component recognizing adhesive matrix molecules (MSCRAMMs) found in Gram-positive bacteria, including staphylococci, enterococci, and streptococci. These molecules share a structural motif consisting of two adjacent immunoglobulin (IgG)-like folded domains at the N-terminal region, which mediates the attachment of MSCRAMMs to host cells or ECM by similar mechanisms (Foster, 2019). Some MSCRAMMs can bind to multiple ligands.

Early electron microscopy studies demonstrated that some *Gardnerella vaginalis* strains possessed pili, which may mediate adherence to human cells (Johnson and Davies, 1984; Boustouller et al., 1987). The extent of pilation depended on the cultivation conditions and frequency of subculturing. Later studies detected the potential loci involved in the pili assembly in the genomes of several *Gardnerella* strains. However, the pili were not identified by electron microscopy (Harwich et al., 2010).

The surface-exposed proteins of *G. vaginalis* strain ATCC 14018 were analyzed for the first time by cell surface shaving and proteomic analysis (Marín et al., 2018). This analysis identified 261 surface-associated proteins, 80 of which contained motifs characteristic of surface-anchored proteins, potentially including adhesins. In this initial approach to identify *Gardnerella* adhesins, we analyzed the surface-exposed protein named Cna by Marín et al. (2018). Its subcellular localization was verified by immunofluorescence assays with monoclonal antibodies generated against recombinant protein (Marín et al., 2018). *G. vaginalis* Cna (Grd Cna) contains the B-type domain characteristic of staphylococcal serine-aspartate repeat-containing Sdr proteins of MSCRAMMs that bind the ECM components, including human fibrinogen (Wang et al., 2013). Sdr proteins share a similar structural organization, comprising an N-terminal secretory signal, a ligand-binding region with two adjacent IgG-like folded N2 and N3 domains, and a B domain composed of repeated subdomains. This is followed by an R region containing Ser-Asp repeats. The C-terminal includes an LPXTG cell wall-anchoring motif, a membrane-spanning region, and a cytoplasmic domain (Wang et al., 2013; Foster, 2019).

In this study, we employed computational modeling to predict the putative structure of Grd Cna protein. The model revealed that the structural organization of Grd Cna resembles that of Sdr and UafA adhesins of the MSCRAMM family. The prevalence of the *cna* gene among *Gardnerella* isolates was also determined. Furthermore, we demonstrated that the recombinant Grd Cna protein containing a putative ligand-binding region can directly bind to fibrinogen.

## 2 Materials and methods

### 2.1 Computational analysis of protein sequence and structure modeling

The sequence of the Grd Cna protein from *G. vaginalis* ATCC 14018 (RefSeq: WP_009994263.1) was analyzed using the InterPro (Paysan-Lafosse et al., 2023), COMER (Margelevičius, 2020; Dapkūnas and Margelevičius, 2023), and HHpred (Zimmermann et al., 2018) servers. Disordered and membrane regions were predicted using the PSIPRED Workbench (Buchan and Jones, 2019). As the structure model for this protein was unavailable in the AlphaFold Database (Varadi et al., 2024), the AlphaFold DB model for a 100% identical protein with eight additional N-terminal residues (UniProt: E3D8G7) was used for initial structural analysis. Identified domains were remodeled using the AlphaFold 2 full-dbs preset (Jumper et al., 2021) to generate more reliable models. For cases where AlphaFold 2 did not produce confident models, additional structure modeling was performed using a ColabFold-based pipeline incorporating multiple sequence alignments from metagenomic data (Olechnovič et al, 2023), RoseTTAFold (Baek et al., 2021), and AlphaFold 3 (Abramson et al., 2024). The selected top models were relaxed by short molecular dynamics simulations using OpenMM (Eastman et al., 2017; Jumper et al., 2021). Additional model evaluation was done using VoroMQA (Olechnovič and Venclovas, 2017) and MolProbity (Williams et al., 2018). Structure-based searches were conducted with the DALI web server (Holm, 2022), and protein structures were compared using TM-align (Zhang and Skolnick, 2005).

To detect the prevalence of *cna*-positive strains among *Gardnerella* spp., the whole genome sequences were downloaded from NCBI Datasets (https://www.ncbi.nlm.nih.gov/datasets/genome/?taxon=2701). Out of 271 entries, metagenome-assembled genomes and assemblies smaller than 1.47 MB were excluded. A single dataset was selected for ATCC 14018 and 14019 strains. The database, comprising 144 unique datasets, was analyzed using TBlastN version 2.16.0+. The query consisted of amino acid residues 27–671 of Cna protein from *G. vaginalis* ATCC 14018, with the search restricted by an E-value threshold of 1E-90.

### 2.2 *Gardnerella* strains and culture conditions

*G. vaginalis* strains ATCC 14018 and 49145 were purchased from the American Type Culture Collection (ATCC). Thirty-four *Gardnerella* isolates were previously characterized and assigned to the species (Bulavaite et al., 2021). Bacterial stocks were frozen at -80°C in tryptic soy broth (Liofilchem) supplemented with 20% horse serum (Oxoid) and 15% glycerol. Each isolate was inoculated from bacterial stock on plates containing chocolate agar with Vitox (Oxoid) and incubated at 37°C in an AnaeroJar (2.5 L) with CO_2_ Gen sachet (Oxoid) for 24–48 h. For adhesion and flow cytometry assays, strains were grown in 7 mL of liquid BHI medium (Liofilchem) supplemented with 1% glucose and 2% horse serum in tightly closed tubes at 37°C for 18 h. Bacteria were collected by centrifugation and washed with serum-free BHI or Dulbecco’s Phosphate-Buffered Saline (DPBS), respectively.

### 2.3 PCR to identify the *cna* gene in *Gardnerella* isolates

Genomic DNA was extracted from *Gardnerella* isolates using the GenJet Genomic DNA Extraction Kit (Thermo Fisher Scientific). The primer sequences and PCR conditions to amplify the full-length *cna* gene are provided in **Supplementary Methods**. To enhance the success of *cna* identification across isolates, we also amplified gene fragments using multiplex PCR as described in **Supplementary Methods**. The purified PCR products were subjected to Sanger sequencing.

### 2.4 Production of recombinant Grd Cna protein

The *cna* gene fragment corresponding to amino acids 27 to 532 was amplified from *G. vaginalis* isolate 114.2 genomic DNA using primers Cna-For and N-cna-Rev (**Supplementary Methods**). The 1518 bp DNA fragment was sequenced and cloned into pET28a(+) vector (Merck Millipore). The resulting plasmid was transformed into *E. coli* Tuner (DE3) strain (Merck Millipore). Protein synthesis was induced with 0.1 mM isopropyl β-D-1-thiogalactopyranoside (IPTG) for 4 h at 26°C. Recombinant Cna (rCna) protein was purified using immobilized metal ion affinity chromatography on Chelating Sepharose Fast Flow with immobilized Ni^2+^ ions (GE Healthcare). The purified protein was aliquoted and stored in a 20 mM HEPES, 0.1 M NaCl, pH 7.5 solution at –80°C.

### 2.5 Generation of antibodies against the Grd rCna protein

All animal experiments were reviewed and approved by the Lithuanian State Food and Veterinary Agency (permission no. G2-117, valid until 30-04-2024). All animal maintenance and experimentation followed FELASA guidelines as well as Lithuanian and European legislation and were conducted at the Life Sciences Center, Vilnius University. Four male 8-week-old BALB/c mice were immunized with 50 µg of rCna by intraperitoneal injection, administered thrice at 28-day intervals. Complete and incomplete Freund’s adjuvants (Sigma Aldrich) were used for the first and second immunizations. The final boost was given to the mouse with the highest antibody titer against rCna. Mice were then sacrificed, and blood samples were collected. Polyclonal antibodies (PAbs) were isolated by a standard procedure, while monoclonal antibodies (MAbs) were produced using the hybridoma technique described in (Kucinskaite-Kodze et al., 2020).

### 2.6 Flow cytometry

*G. vaginalis* ATCC 14018 cells were stained with CFDA-SE dye (Bio-Rad) and exposed to mouse polyclonal and monoclonal antibodies targeting the rCna protein. Detection was performed using Alexa Fluor 647-conjugated goat anti-mouse IgG antibodies (Thermo Fisher Scientific). CFSE fluorescence was measured with a 488 nm laser and 530/30 filter on a BD FACSymphony A1 flow cytometer (BD Biosciences). Alexa Fluor 647 fluorescence was detected using a 637 nm laser and 670/30 filter. Data were analyzed and visualized using FlowJo software (v10, BD Biosciences). See **Supplementary Methods** for details.

### 2.7 Enzyme-linked immunosorbent assay (ELISA)

Human fibrinogen (Fbg), fibronectin (Fn), and collagen (Col) type I, type III, and type IV were purchased from Sigma-Aldrich. A 96-well plate was coated with ECM proteins (2-3 µg/µL per well). The rCna solution containing 0.02-1 µg protein/well was incubated with immobilized proteins. Complex formation was detected with anti-Cna MAbs followed by the addition of an anti-mouse IgG antibody conjugated with horse-radish peroxidase. See **Supplementary Methods** for details.

### 2.8 *G. vaginalis* adherence to immobilized fibrinogen

Human ECM proteins were immobilized on 96-well microtiter plates, with human serum albumin (HSA) as a negative control. Coating, blocking, and washing conditions followed the ELISA protocol (**Supplementary Methods**). Fifty-µL of *G. vaginalis* ATCC 14018 cell suspension in serum-free BHI (OD_600_ = 0.2) was added to each well. Plates were incubated in a Compact plastic pouch (Thermo Fisher Scientific) with a CO_2_ Gen sachet for 24 h at 37°C. Unattached bacteria were removed by decanting, the wells were rinsed with phosphate-buffered saline (PBS) twice and dried at 37°C for 20 min. Adherent bacteria were stained with 50 µL of a safranin solution (Sigma-Aldrich) per well for 10 min at 37°C. After three PBS rinses, the wells were dried at 37°C. The safranin stain was then solubilized by adding 50 µL of 33% acetic acid per well, and adhesion was quantified by measuring optical density at 527 nm with a reference wavelength at 620 nm.

## 3 Results

### 3.1 Structural organization of Grd Cna

**Figure 1:**
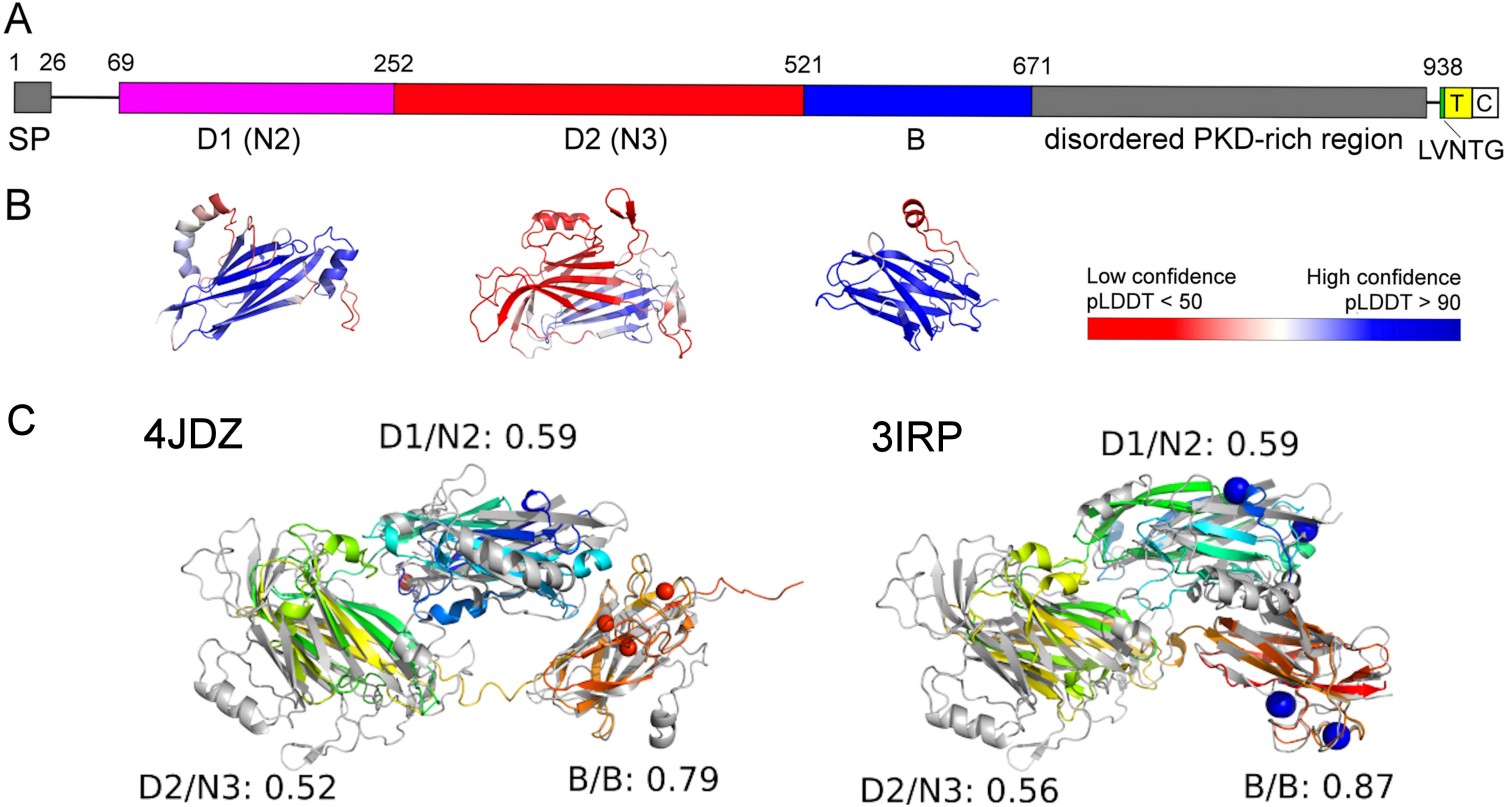
Domain organization of the Cna protein based on sequence analysis and structure modeling. **(A)** A schematic representation of the full-length Grd Cna protein shows a signal sequence (SP) followed by a putative ligand binding region comprising of domains N2 and N3, a B domain, a PKD-rich region, a cell wall sorting signal (LVNTG), a transmembrane domain (T), and a cytoplasmic domain (C). **(B)** Predicted structures of the respective N2, N3, and B domains colored according to AlphaFold2’s confidence self-estimation scores (predicted local distance difference test, pLDDT). **(C)** Structural alignment of domain models with the corresponding domains of PDB structures 4JDZ and 3IRP. Domain models are shown in gray, while experimental structures are depicted in a rainbow color scheme, transitioning from the N-terminus (blue) to the C-terminus (red). Alignments were generated using TM-align, with TM-scores normalized to the domain of the experimental structure.

The protein sequence analysis using remote homology search tool COMER revealed that Grd Cna contains the SdrD_B domain (Pfam: PF17210) spanning amino acid residues 521-667. According to PSIPRED Workbench, the N- and C-terminal regions are likely disordered, with a transmembrane region at the C-terminus adjacent to the non-canonical signature motif LVNTG (**Figure 1**; **Supplementary Table S1**). Sequence-based searches did not identify proteins with known experimentally determined structures.

In the AlphaFold database model (https://alphafold.ebi.ac.uk/entry/E3D8G7), the SdrD_B domain is predicted with high confidence (residues 521-671). Another high-confidence domain is predicted near the N-terminus (residues 69-252) and is hereafter referred to as domain 1 (D1) in this study. These two domains are connected by an extended region, termed D2, whose structure was predicted with very low reliability. We remodeled these three domains separately for a more detailed structural and functional analysis.

Structure modeling using AlphaFold2 was successful only for the D1 and SdrD_B domains (**Supplementary Table 1**). The N-terminal region (residues 1–530) had a very low number of homologs in protein sequence databases. To address this, we explored metagenomic sequence databases to identify additional homologs and improve structure prediction for the D2. However, these efforts were unsuccessful. As an alternative, we submitted the D2 sequence to the AlphaFold Server, which utilizes AlphaFold3, the latest and fully re-trained AlphaFold version. AlphaFold3 produced a model with higher confidence, featuring a predicted TM-score of 0.55. While this score suggests only moderate reliability, it indicates that the model could potentially provide a correct fold prediction (Xu and Zhang, 2010). Thus, we included this model in our further analyses. Additionally, VoroMQA statistical potential scores, as well as MolProbity-based analysis, indicate high quality for the structural models of all domains (**Supplementary Table S1** and **Supplementary Figure S1**).

We submitted the structural models of all three domains to the DALI server to identify potential remote homologs with similar functions. For the SdrD_B domain, DALI identified three PDB entries with Z-scores > 14: 4JDZ, 8VDK, and 3IRP (Wang et al., 2013; Matsuoka et al., 2011) (**Supplementary Table S2**). These proteins are cell wall-anchored proteins from *Staphylococcus aureus* and *Staphylococcus saprophyticus*, each containing three domains with an IgG-like fold (Schaeffer et al., 2024). Interestingly, when the D1 structure model was used as a query among multiple bacterial cell surface-anchored proteins, the same PDB entries were also identified, with the D1 model aligning to the N2 domain of these structures. When the D2 model of lower confidence was used, DALI identified different PDB entries, many of which were bacterial cell surface proteins containing IgG-like domains. Yet, the structural models of all three domains align well with the corresponding N and B domains of PDB structures for *S. aureus* SdrD (4JDZ) and *S. saprophyticus* UafA (3IRP), achieving TM-scores > 0.5 (**Figure 1C**). Based on sequence analysis and structure modeling, we conclude that the protein is likely a cell surface protein with two N domains, each resembling an IgG-like fold (Deivanayagam et al., 2002) and one B domain.

### 3.2 Prevalence of the *cna* gene among *Gardnerella* isolates

We tested the presence of the *cna* gene by PCR across 34 isolates, specifically *G. vaginalis* (n=15), *G. piotii* (n=6), *G. pickettii* (n=4), *G. swidsinskii* (n=3), and *G. leopoldii* (n=6). The *cna* gene was detected in five isolates—76.2, 84.5, 105.1, 106.5, and 114.2—all classified as *G. vaginalis* (**Supplementary Figure S2**). A multiple alignment of the deduced amino acid sequences of Grd Cna from the isolates revealed high homology in the region spanning N2-N3 and B domains (**Supplementary Figure S3**). The PKD-rich region was the most variable across isolates, showing deletions and insertions. To assess the prevalence of the *cna* gene across diverse *Gardnerella* strains, we analyzed publicly available genomes from the NCBI Genome database. This analysis included 144 genomes of clinical *Gardnerella* isolates, using the highly homologous Cna region consisting of N2-N3 and B domain as a query. We identified the Grd Cna protein in 38 strains (26.4%): four classified as *Gardnerella* genome species 2, two as genome species 7, thirty-one as *G. vaginalis*, and one as *Gardnerella* spp.

### 3.3 Expression of Cna on *G. vaginalis* cell surface

Flow cytometry analysis was used to visualize Cna expression on *G. vaginalis* ATCC 14018 cell surface. Cells were stained with a fluorescent dye CFDA-SE, incubated with MAbs or PAbs raised against rCna, and subsequently treated with Alexa Fluor 647-labeled goat anti-mouse IgG. Anti-rCna MAbs demonstrated increased fluorescence intensity of *G. vaginalis* cells compared to the isotype control, indicating specific binding to the Grd Cna protein (**Supplementary Figure S4**). The proportion of Alexa Fluor 647-positive cells ranged from 29.9% to 31.6% across the tested anti-rCna MAbs (**Supplementary Figure S4E**). Anti-rCna PAbs showed significantly higher fluorescence intensity, suggesting strong and specific binding (**Supplementary Figure S4F**). The frequency of Alexa Fluor 647-positive cells ranged from 33.7% to 60.5%, whereas irrelevant PAb exhibited only 25.6% positivity, indicating minimal nonspecific binding. Two distinct peaks were observed within the CFDA-SE-stained *G. vaginalis* cell population (**Supplementary Figures S4E, F**). The first peak, characterized by near zero Alexa Fluor 647 fluorescence, indicated the absence of interaction between Grd Cna and antibody and likely represented bacterial cells lacking surface-exposed Cna. The second peak with high fluorescence intensity indicated the presence of Grd Cna on the bacterial cell surface. The application of Grd Cna-targeting polyclonal antibodies produced high-fluorescence peaks with varying intensities, reflecting their differential binding affinities to Cna (**Supplementary Figure S4F**). Western blot analysis detected Grd Cna in the cell wall extracts of *G. vaginalis* strains 114.2 and ATCC 14018 but not in the *cna*-negative strain ATCC 49145 (**Supplementary Methods** and **Supplementary Figure S5**).

### 3.4 Binding of rCna to fibrinogen

We tested the interaction of the putative ligand binding region of Cna derived from *G. vaginalis* strain 114.2 with ECM components, specifically Col I, Col III, Col IV, Fbg, and Fn. The amino acid identity of this region is 99.4% between *G. vaginalis* strains ATCC 14018 and 114.2. The region comprising 506 amino acids was expressed in *E. coli* with the N-terminal His tag (calculated molecular weight 59 kDa). The purity of the rCna protein was confirmed by SDS-PAGE analysis, showing migration at an apparent molecular weight of 60 kDa (**Supplementary Figure S6**). The stability of rCna was assessed using a fluorescence-based thermal shift assay (**Supplementary Methods**). In this assay, the rCna protein displayed a single sharp unfolding transition at approximately 50°C, characteristic of single-domain globular proteins (Gao et al., 2020). The protein demonstrated stability across a pH range of 3.5 to 8.5.

**Figure 2:**
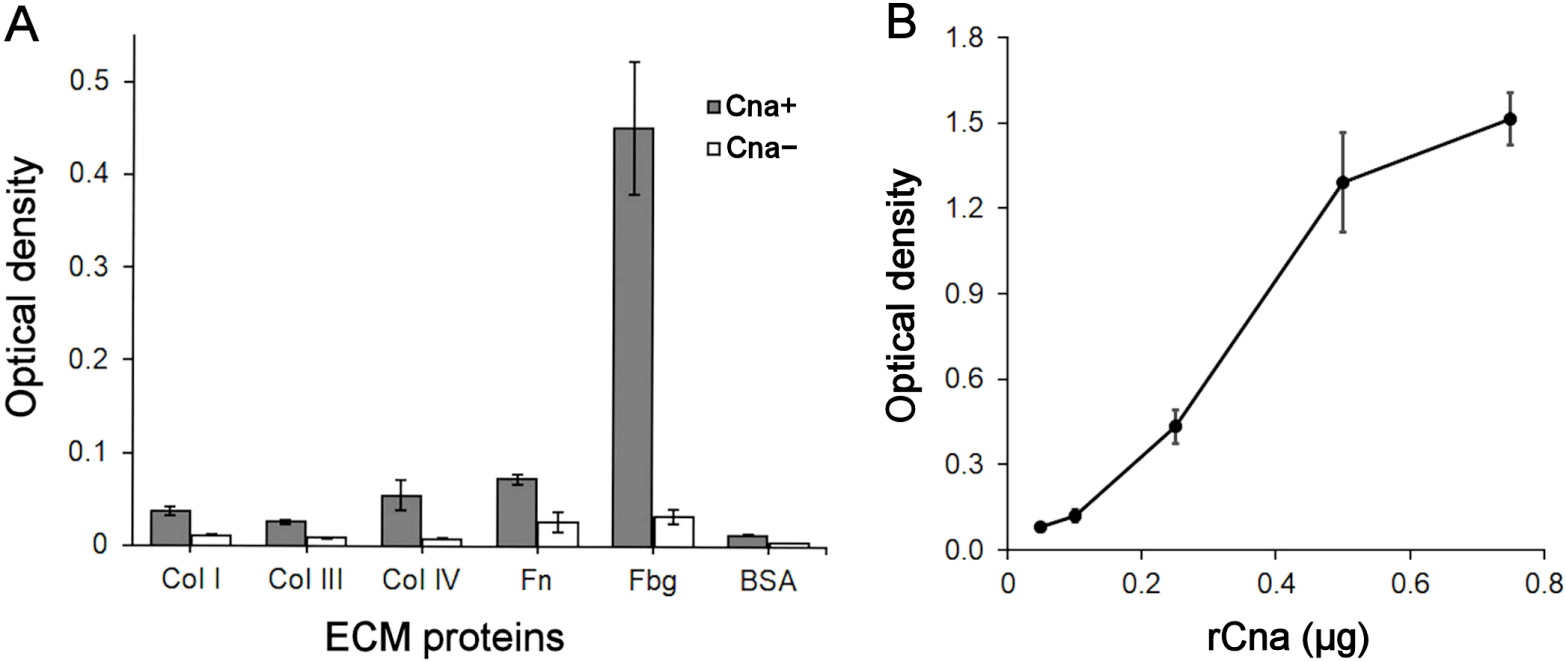
Interaction of Grd rCna with human ECM components. **(A)** ELISA was performed with collagen types I, III, IV, Fn, Fbg, and BSA immobilized on microtiter plates. Each well was incubated with either 0.25 µg/well of rCna (Cna+) or no rCna (Cna−). Bound rCna was detected with anti-rCna MAb clone E2 (0.1 µg/well). Data are presented as the means ± SD from two independent experiments, each with four technical replicates. **(B)** ELISA showing dose-dependent binding of rCna to Fbg. Increasing concentrations of rCna (0.05, 0.1, 0.25, 0.5, and 0.75 µg/well) were used, and bound rCna was detected with the anti-rCna MAb clone E2 (0.1 µg/well). Data are presented as the means ± SD from two independent experiments, each with four technical replicates.

The ECM components immobilized on microtiter plates were incubated with rCna, and protein binding was evaluated using customized MAbs against rCna. Specific binding of rCna to Fbg was detected but not to the collagen types or Fn (**Figure 2A**). When Fbg was preincubated with varying amounts of rCna, a dose-dependent binding to the immobilized Fbg was observed (**Figure 2B**).

**Figure 3:**
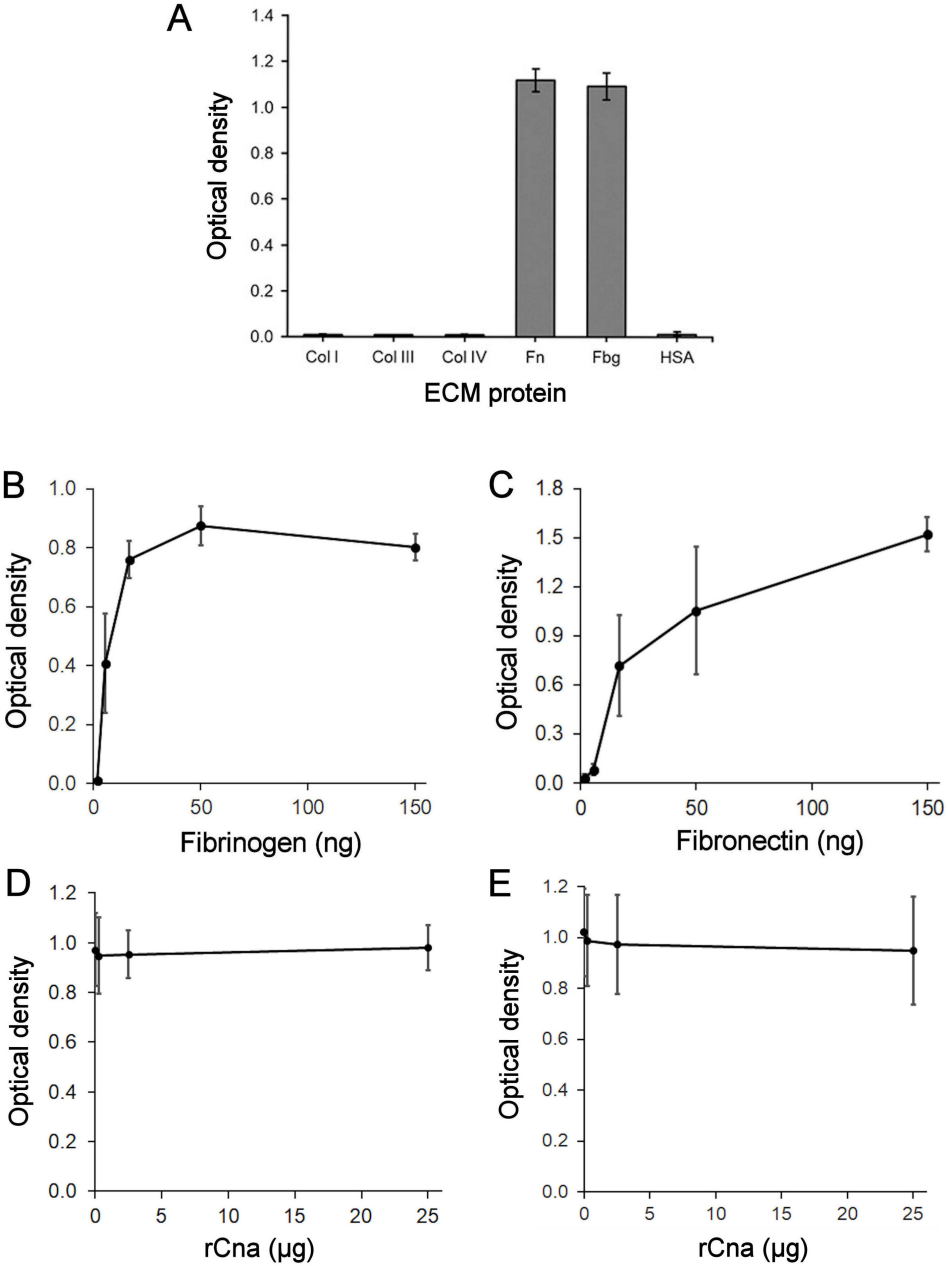
Binding of Cna-positive *G*. *vaginalis* ATCC 14018 to human ECM components. ECM proteins and human serum albumin (HSA) were immobilized on microtiter plates at a concentration of 125 ng/well **(A).** Fibrinogen **(B)** or fibronectin **(C)** were immobilized at concentrations 1.85, 5.56, 16.67, 50, and 150 ng/well, and incubated with *G. vaginalis* cells in serum-free BHI medium. Fibrinogen (3 µg/well) **(D)** or fibronectin (2 µg/well) **(E)**-coated plates were incubated with 0.25, 2.5, and 25 µg/well of rCna before adding *G*. *vaginalis*. Non-adherent cells were removed, while adherent bacteria were stained with safranin. Data were presented as the means ± SD from two **(A–C)** or three **(D, E)** independent experiments with four **(A)** or six **(B–E)** technical replicates.

We further evaluated the adhesion of the Cna-positive *G. vaginalis* ATCC 14018 strain to ECM proteins. No attachment to collagen types I, III, or IV was detected (**Figure 3A**). Bacterial cells were incubated with varying amounts of immobilized Fbg or Fn. The attached bacteria were quantified by staining. *G. vaginalis* demonstrated effective, dose-dependent adherence to Fbg and Fn (**Figures 3B, C**). While rCna showed specific binding to Fbg (**Figures 2A, B**), increasing concentrations of rCna did not inhibit *G. vaginalis* binding to immobilized Fbg (**Figure 3D**). rCna did not affect *G. vaginalis* adherence to Fn (**Figure 3E**) and it did not bind to immobilized Fn (**Figure 2A**). The same rCna preparation was used for binding and inhibition experiments.

## 4 Discussion

*Gardnerella* is a component of the vaginal microbiota in women with BV; however, it is also present in healthy women, typically in lower numbers (Janulaitiene et al., 2017). Extensive vaginal colonization by *Gardnerella*, following the establishment of a polymicrobial biofilm, is a hallmark feature of BV (Muzny et al., 2019; Chen et al., 2021). To date, little is known about the factors that enable *Gardnerella* to adhere to host cells facilitating its colonization and persistence in the vaginal environment.

The computational analysis of protein sequence and structure modeling revealed that the surface-exposed Grd Cna protein resembles the structure of MSCRAMMs of Gram-positive cocci (Foster, 2019). The putative ligand-binding region of Gdr Cna consists of two domains, N2 and N3, which resemble an IgG-like fold and exhibit structural homology to the corresponding domains in the SdrD and UafA proteins (Wang et al., 2013; Matsuoka et al., 2011). The Sdr proteins are a part of the archetypal Clf-Sdr-FnBP family of MSCRAMMs, which consist of three N-terminal domains (N1, N2, and N3). However, ligand binding, including binding to Fbg, is mediated exclusively by the N2 and N3 domains through the “dock, lock, and latch” (DLL) mechanism (Deivanayagam et al., 2002; Wang et al., 2013). Notably, the ligand binding region of *Streptococcus agalactiae* Srr proteins typically includes only N2 and N3 homologs that participate in Fbg-binding by the DLL mechanism (Foster, 2019). In contrast, the active involvement of both B and N2-N3 domains is essential for the UafA protein to bind to its ligand (Matsuoka et al., 2011).

The Grd Cna protein includes the B domain characteristic of the Sdr group of adhesins in Gram-positive cocci (Foster, 2019). The number of B domains in MSCRAMMs varies across proteins: for instance, the *S. saprophyticus* adhesin UafA contains a single B domain downstream of three N domains (Matsuoka et al., 2011), while two or more separately folded B domains are present in Sdr proteins (Ajayi et al., 2018; Foster, 2019). The functions of B domains in the Clf-Sdr-FnBP proteins remain elusive, while the B domain in UafA participates in ligand binding (Matsuoka et al., 2011).

Downstream of the B domain, the R region of Grd Cna contains a disordered PKD-rich region, which, to the best of our knowledge, has not been identified in other adhesins. The R region of Clf-Sdr-FnBP proteins is enriched in Ser-Asp repeats, UafA features a Ser-Glu-rich region, and *S. agalactiae* adhesin PbsP contains a Met-Lys-rich region in its distal part. Notably, the length of the R region varies among *Gardnerella* isolates, a trait also observed in other MSCRAMMs (Ajayi et al., 2018). The R region of the Clf-Sdr family proteins functions as a stalk, projecting the ligand-binding domain away from the cell surface to facilitate the conformational changes necessary for effective ligand binding (Foster, 2019). Although the Grd Cna protein carries a nontypical LVXTG sorting signal in the C-terminal, it was demonstrated that sortases can display proteins with variations of sequence motif on the cell surface (Ton-That et al., 2004).

A recombinant Grd Cna protein containing a putative ligand-binding region, which includes the N2 and N3 domains, specifically bound to Fbg *in vitro* in a dose-dependent manner but showed no binding to Fn or collagen types I, III, and IV. Flow cytometry and Western blot analysis revealed that *G. vaginalis* ATCC 14018 cells produce Cna, which is localized on the cell surface. However, flow cytometry analysis demonstrated that a substantial proportion of *G. vaginalis* cells did not interact with Cna-specific antibodies. This observation suggests that phase-variable colony variants within the *G. vaginalis* population can contribute to heterogeneity, potentially leading to differential expression of proteins, including virulence factors (Garcia et al., 2024). We demonstrated that Cna-positive *G. vaginalis* cells adhered to immobilized Fbg. The observed *G. vaginalis* cell adherence to Fn suggests that additional cell-surface components of *Gardnerella* function as adhesins. However, when immobilized Fbg was preincubated with varying amounts of rCna, no dose-dependent inhibition of *G. vaginalis* adhesion to Fbg was observed. These results demonstrate that, under the tested conditions, Grd Cna is not a major cell-surface component mediating *Gardnerella* adherence to Fbg.

Fbg is a highly abundant protein in human plasma and also serves as a component of ECM. Fbg is detected in vaginal lavage fluid and it is produced by ecto- and endocervical cell lines derived from the human female genital tract, which immobilize it on their cell surfaces (Wang et al., 2014). However, during *Gardnerella* colonization, the mucous layer covering vaginal epithelial cells may mask the availability of Fbg. Sialidases, abundantly produced by *Gardnerella* and, particularly, *Prevotella*, degrade the protective mucous layer in the cervicovaginal environment (Pelayo et al., 2024), thereby facilitating bacterial adherence through interactions between bacterial adhesins and their corresponding ligands. The interaction with human Fbg has also been detected in the Gram-positive bacterium *S. agalactiae*, a common vaginal colonizer and a significant cause of invasive disease in neonates (Buscetta et al., 2014). Multiple structurally distinct Fbg-binding proteins have been identified in *S. agalactiae*, enabling the bacterium to facilitate colonization and penetrate host barriers.

Interestingly, the *cna* gene coding for Grd Cna was predominantly detected in *G. vaginalis* isolates but was absent in isolates of other commonly detected vaginal *Gardnerella* species, including *G. piotii*, *G. swidsinkii*, and *G. leopoldii*. However, the limited number of isolates analyzed in this study, both *in silico* and experimentally, may have hindered the determination of the true prevalence of this gene among *Gardnerella* species. Notably, the studies have demonstrated that *G. vaginalis* is strongly associated with BV, while other *Gardnerella* species show varying associations with the disease (Hill et al., 2019).

## 5 Conclusion

The pathogenesis of *Gardnerella* remains an important yet understudied area of research. This study presents an initial analysis of the surface-associated Grd Cna protein, with computational structure modeling and analysis revealing its similarity to the staphylococcal Sdr and UafA adhesins of the MSCRAMM family. We provide evidence that recombinant Grd Cna binds fibrinogen in a dose-dependent manner and that Cna-positive *G. vaginalis* cells adhere to fibrinogen *in vitro.* Further studies are needed to identify other specific *Gardnerella* determinants involved in this interaction, as our findings suggest that Grd Cna may not be a major adhesin mediating *G. vaginalis* adherence to fibrinogen.

## Data Availability

The datasets presented in this study can be found in online repositories. The names of the repository/repositories and accession number(s) can be found below: https://www.ncbi.nlm.nih.gov/genbank/, PP874905– PP874907.
