# Supplementary material for: Gardnerella fibrinogen-binding protein as a candidate adherence factor
Source: Front Cell Infect Microbiol. 2025 May 8;15:1556232. doi: 10.3389/fcimb.2025.1556232 (PMC12095359; doi:10.3389/fcimb.2025.1556232)
Supplement: Supplementary file 1 [file DataSheet1.pdf]

Supplementary Material

Gardnerella fibrinogen-binding protein as a candidate adherence factor

Aistė Bulavaitė, Justas Dapkūnas, Raminta Reškevičiūtė, Indrė Dalgėdienė, Lukas Valančauskas, Lina Baranauskienė, Milda Plečkaitytė*

*** Correspondence:** Corresponding Author: milda.pleckaityte@bti.vu.lt

1 Supplementary Methods

1.1 PCR conditions for amplification of the *cna* gene

Amplification of the full-length *cna* gene was performed using Hot Start DreamTaq PCR Master Mix (Thermo Fisher Scientific) and the primers Cna-For and Cna-Rev (Table SM1). The reaction mixture was subjected to initial denaturation at 94°C for 2 min followed by 35 cycles of denaturation at 94°C for 20 s, primer annealing at 50°C for 20 s, and extension at 72°C for 1 min 30 s. These were followed by a final extension step for 1 min at 72°C.

The *cna* gene fragment corresponding to amino acids 27 to 532 was amplified from *G. vaginalis* isolate 114.2 genomic DNA using Phusion Flash High Fidelity PCR Master Mix (Thermo Fisher Scientific) and the primers Cna-For and N-cna-Rev. The reaction mixture was subjected to initial denaturation at 98°C for 10 s followed by 30 cycles of denaturation at 98°C for 5 s, primer annealing at 62°C for 10 s, and extension at 72°C for 20 s. A final extension step lasted for 1 min at 72°C.

Table SM1. Primers used for amplification of the *cna* gene

Primer	Sequence (5′→3′)
Cna-For	TCGCTAGCCAGTCGAGCAATGATAATGCTT
Cna-Rev	AACTCGAGTTATTGCCTGTGCTTGCC
N-cna-Rev	TTCTCGAGTTAGTTAGCATCAAACCACACGC
Cna-Sdr-For	GTGTGGTTTGATGCTAACCAT
Cna-Sdr-Rev	CTTAAAAGCTCCAGCATCAATATGC

A PCR to explore the presence of the *cna* gene was performed using the primers Cna-For and N-cna-Rev. The reaction mixture containing Hot Start DreamTaq PCR Master Mix was subjected to initial denaturation at 94°C for 2 min followed by 35 cycles of denaturation at 94°C for 20 s, primer annealing at 50°C for 20 s, and extension at 72°C for 1 min 30 s. The PCR products were analyzed using agarose gel electrophoresis (Figure S3).

A multiplex PCR was also performed to amplify the *cna* gene fragments using the primers Cna-Sdr-For, Cna-Sdr-Rev, Cna-For, and N-cna-Rev (Table SM1). The amplification mixture prepared using

Phusion U Multiplex PCR Master Mix (Thermo Fisher Scientific) was subjected to initial denaturation at 98°C for 30 s, and 34 cycles of denaturation for 10 s at 98°C, annealing for 30 s at 61°C, and extension for 1 min at 72°C.

1.2 ELISA

ECM proteins were dissolved in either 0.05 mol/L $NaHCO_3$ (pH 9.5) for collagen types I, III, IV (Col I, Col III, Col IV), and fibronectin (Fn) or in phosphate-buffered saline (PBS) for fibrinogen (Fbg). A 96-well unmodified polystyrene plate (Nerbe plus) was coated with 50 µL per well of Col I, Col III, or Fn solution. For Col IV and Fbg, 50 µL per well was coated on a modified polystyrene plate (Polysorp, Thermo Fisher Scientific). The protein concentration per well was 2 µg for Col I, Col IV, and Fn, and 3 µg for Col III and Fbg. Plates were incubated overnight at 4°C for Col I, Col III, Col IV, and Fn, or 2 hours at 37°C on a plate shaker for Fbg. Following incubation, plates were washed twice with PBS supplemented with 0.1% Tween-20 (PBST). Wells were then blocked with 250 µL of 2% bovine serum albumin (BSA) in PBS for 1 hour at room temperature (RT) on a plate shaker. After two additional washes with PBST, 50 µL of rCna solution in PBST (0.02–1 µg protein/well) was added to each well, and the plates were incubated for 1 hour at RT. Following four washes with PBST, 50 µL of MAb solution in PBST was added to each well, and the plates were incubated for 1 hour at RT. After another four washes, 50 µL of goat anti-mouse IgG antibody conjugated with horseradish peroxidase (HRP) (1:5000 dilution, Bio-Rad) was added to each well, and the plates were incubated for 1 hour at RT. Plates were then washed seven times with PBST followed by two washes with deionized water. The enzymatic reaction was initiated by adding 50 µL of 3,3′,5,5′-tetramethylbenzidine (TMB; Clinical Science Products) to each well. After 15 minutes at RT, the reaction was stopped with 25 µL of 3.6% H_2SO_4. Optical density (OD) was measured at 450 nm with a reference wavelength of 620 nm using a Multiscan GO microtiter plate reader (Thermo Scientific).

1.3 Flow cytometry analysis

G. vaginalis ATCC 14018 cells were cultivated in BHI medium (Liofilchem) supplemented with 1% glucose and 2% horse serum at 37°C for 18 h. The cells were then washed with Dulbecco's Phosphate-Buffered Saline (DPBS), and divided into the test and negative control samples. Test samples were stained with 5 µM carboxyfluorescein diacetate succinimidyl ester (CFDA-SE) (Bio-Rad), prepared by diluting 25 µL CFDA-SE in 975 µL DPBS. Staining was performed at RT for 20 min with gentle rotation. CFDA-SE forms fluorescent carboxyfluorescein succinimidyl ester (CFSE) inside the cells. Following labeling, the cells were washed once with DPBS and twice with fluorescence-activated cell sorting (FACS) buffer. CFSE-labeled target cells were suspended in 80–100 µL of FACS buffer per sample. Five micrograms of the primary target antibody (MAb or PAb), mouse IgG2b (MAb isotype control), or irrelevant PAb were added to each sample. The samples were incubated at 4°C for 30 minutes in the dark, with gentle agitation for 15 minutes to ensure uniform antibody distribution.

The samples were then washed three times by centrifugation at 3000 × g for 5 minutes at 4°C and resuspended in FACS buffer. Secondary antibodies, Alexa Fluor 647 goat anti-mouse IgG (H+L) (Thermo Fisher Scientific), were diluted in FACS buffer at a 1:200 ratio for PAb and 1:100 for MAb. A 100 µL aliquot of the prepared antibody solution was added to each sample, followed by a 30-minute incubation at 4°C in the dark. Samples were washed twice as described above. Negative control samples underwent identical processing, including all washing, centrifugation, and incubation steps, to account for nonspecific binding and ensure consistency across experimental conditions.

The samples were analyzed using a BD FACSymphony A1 flow cytometer (BD Biosciences). CFSE fluorescence was measured using a 530/30 BP filter with excitation from a 488 nm laser, while Alexa Fluor 647 fluorescence was measured using a 670/30 BP filter with excitation from a 637 nm laser. A minimum of 80,000 events per sample were recorded. Data analysis and visualization were performed using FlowJo software (v10, BD Biosciences). Cells were initially selected based on FSC-A vs. SSC-A parameters. Singlet cells were identified by gating on FSC-A vs. FSC-H and SSC-A vs. SSC-H to exclude doublets, and the analysis focused exclusively on singlet cells. CFDA-SE-positive cells were identified using bisector gates established from unstained bacterial controls, with 99% of unstained cells positioned on one side of the gate. For CFDA-SE-stained samples, cells positioned to the right of the gate were classified as CFDA-SE-positive. For CFDA-SE-positive cells, the fluorescence of Alexa Fluor 647-conjugated secondary antibodies was assessed. Bisector gates were established using isotype control samples, following the same gating strategy used for CFDA-SE. Experimental samples were then analyzed, and antibody activity was evaluated relative to nonspecific binding observed in isotype control or irrelevant PAb samples.

1.4 Fluorescence-based thermal shift assay

The stability of the rCna protein was assessed using a fluorescence-based thermal shift assay (FTSA), also known as differential scanning fluorimetry (Pantoliano et al., 2001; Simeonov, 2013). The protein was heated at a constant rate of 1°C per minute, and unfolding was monitored using extrinsic fluorescence of the 8-anilino-1-naphthalene sulfonate dye. The assays were performed in the QIAGEN Rotor-Gene Q instrument. Fluorescence intensity was monitored in the blue channel using an excitation wavelength of 365±20 nm and a detection wavelength of 460±15 nm, with fluorescence measurement taken every 30 s. The purified rCNA protein at a concentration of 0.4 mg/mL was prepared in 75 mM of the appropriate buffer containing 250 mM NaCl and 5 mM mercaptoethanol. Subsequently, 100 µM of 8-anilino-1-naphtalene sulfonate dye was added. The pH stability of the protein was assessed across a pH range of 3.5 to 8.5 using the following buffers: acetate, MES (2-(N-morpholino)ethanesulfonic acid), HEPES (4-(2-hydroxyethyl)piperazine-1-ethanesulfonic acid), phosphate, and bicine. Fluorescence intensity curves were generated to determine the melting temperature (T_m), defined as the inflection point of the melting curve representing the temperature at which the protein is half-folded and half-unfolded. The T_m values were calculated using the Thermott Webserver (Gedgaudas et al., 2022). The results showed that the unfolding of rCna protein produced a single transition curve across the tested pH range (Figure SM1A). The protein demonstrated the highest thermal stability within the pH range of 4.5 to 7, with maximum stability observed around pH 5.5 (Figure SM1B).

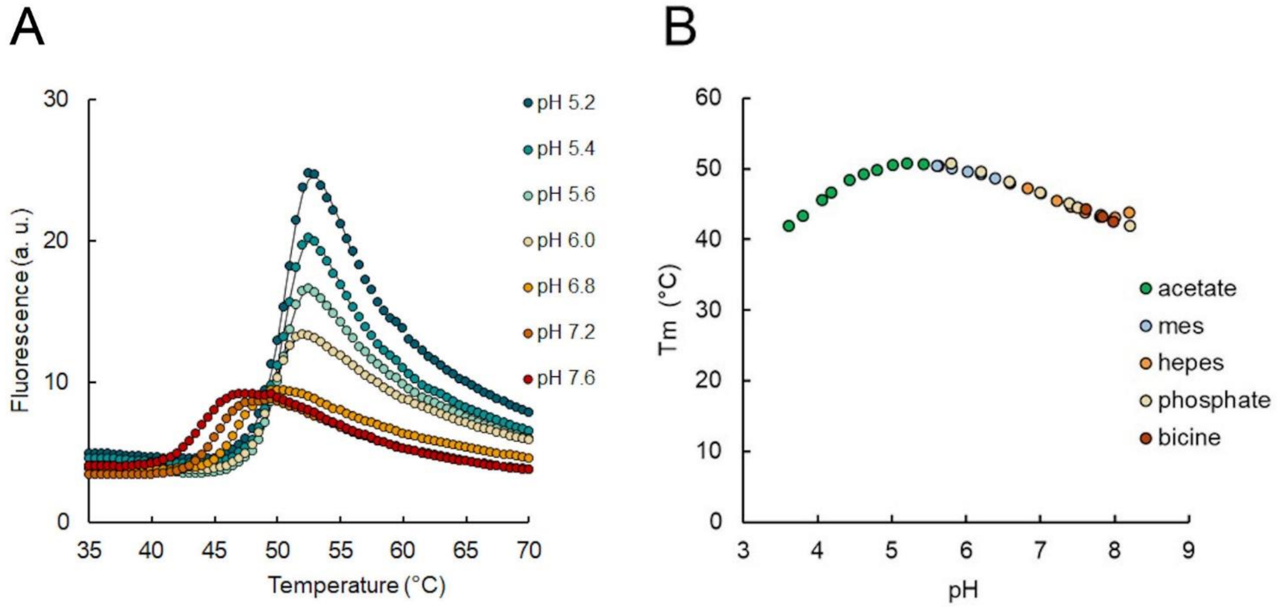

Figure SM1. Analysis of the stability of the rCna protein by FTSA. (**A)** Unfolding curves of rCna in buffers at different pH. (B) Dependence of rCna protein melting temperature (T_m) on pH.

References:

Gedgaudas, M., Baronas, D., Kazlauskas, E., Petrauskas, V., and Matulis D. (2022). Thermott: A comprehensive online tool for protein–ligand binding constant determination. *Drug Discov. Today* 27, 2076-2079.doi: 10.1016/j.drudis.2022.05.008.

Pantoliano, M. W., Petrella, E. C., Kwasnoski, J. D., Lobanov, V. S., Myslik, J., Graf, E., et al. (2001). High-density miniaturized thermal shift assays as a general strategy for drug discovery. *J. Biomol. Screen* 6, 429–440.

Simeonov, A. (2013). Recent developments in the use of differential scanning fluorometry in protein and small molecule discovery and characterization. *Expert Opin. Drug Discov.* 8, 1071–1082.

1.5 Western blot

Bacterial cell wall extracts were prepared using lysozyme (Serva, Germany) in an osmoprotective buffer, as described by Garibaldi et al. (2010). Proteins were separated by SDS-PAGE in a 10% polyacrylamide gel and transferred onto an Immobillon-P PVDF membrane (Merck Millipore). The membrane was blocked with 1% milk powder (Carl Roth, Germany) in PBS at 4°C overnight, followed by four washes with PBST. Next, the membrane was incubated for 1 h at room temperature (RT) with anti-Cna monoclonal antibody (MAb) diluted in PBST containing 1% milk powder, followed by four washes. It was then incubated for 1 h at RT with anti-mouse IgG-HRP (Bio-Rad, USA) in PBST with 1% milk powder, followed by six washes. The membrane was developed using 4-chloro-1-naphthol (Sigma Aldrich, St. Louis, MO, USA) and 3,3',5,5'-tetramethylbenzidine (TMB) NeA-Blue Precip (Clinical Science Products, USA).

Garibaldi M., Rodríguez-Ortega M. J., Mandanici, F., Cardaci, A., Midiri, A., Papasergi, et al. (2010). Immunoprotective activities of a Streptococcus suis pilus subunit in murine models of infection. Vaccine 28, 3609-3616. doi: 10.1016/j.vaccine.2010.01.009

2 Supplementary Figures and Tables

2.1 Supplementary Tables

Supplementary Table 1. Evaluation of protein domain structure models based on AlphaFold's self-estimated accuracy (predicted LDDT and predicted TM-score), VoroMQA and MolProbity

**Domain**	**Structure prediction method**	**pLDDT**	**pTM**	**VoroMQA**	MolProbity score (percentile)
N2 (residues 69-252)	ColabFold, default MSA	75.1	0.74	0.52	0.90 (100th)
N3 (residues 253-520)	AlphaFold 3	51.0	0.55	0.48	0.79 (100th)
B (residues 521-671)	AlphaFold 2, full-dbs preset	86.2	0.84	0.43	1.21 (99th)

Supplementary Table 2. The DALI search identifies MSCRAMMs family proteins as remote homologs of the Cna protein domains

**Domain**	**DALI result PDB ID**	**DALI result no.**	**DALI Z-score**	**RMSD**	**Annotation**
N2 (residues 69-252)	4JDZ (chain A)	327	6.4	3.2	SER-ASP RICH FIBRINOGEN/BONE SIALOPROTEIN-BINDING
N2 (residues 69-252)	3IRP (chain X)	306	6.5	2.9	URO-ADHERENCE FACTOR A
B (residues 521-671)	4JDZ (chain A)	1	14.9	2.0	SER-ASP RICH FIBRINOGEN/BONE SIALOPROTEIN-BINDING
B (residues 521-671)	3IRP (chain X)	3	14.7	1.9	URO-ADHERENCE FACTOR A

2.2 Supplementary Figures

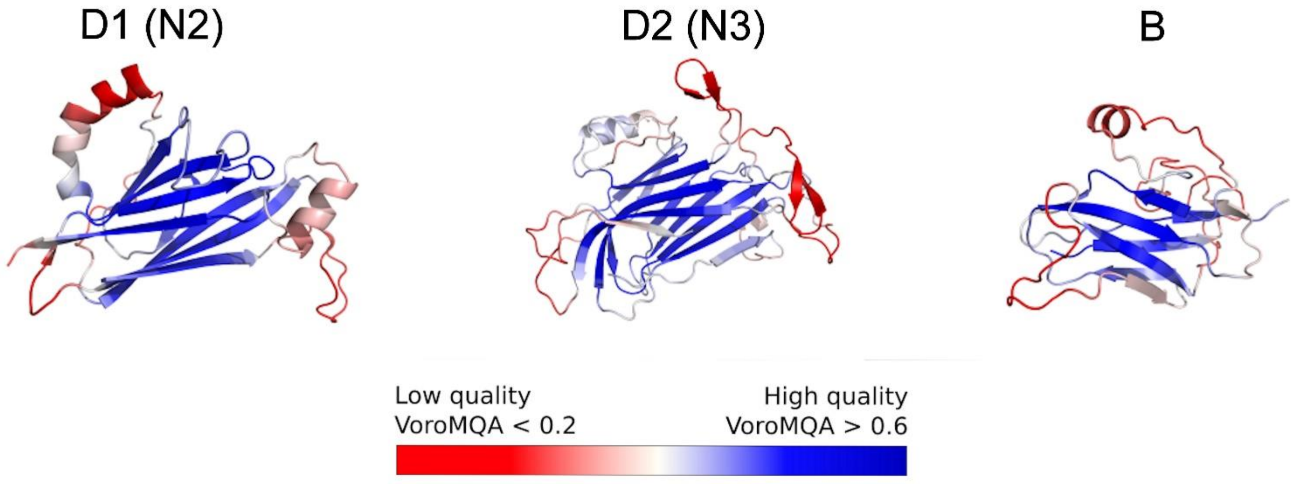

Supplementary Figure 1. Models of the three *Cna* domains (N2, N3, and B), colored based on VoroMQA scores, where red indicates low-quality predictions and blue indicates high-quality predictions.

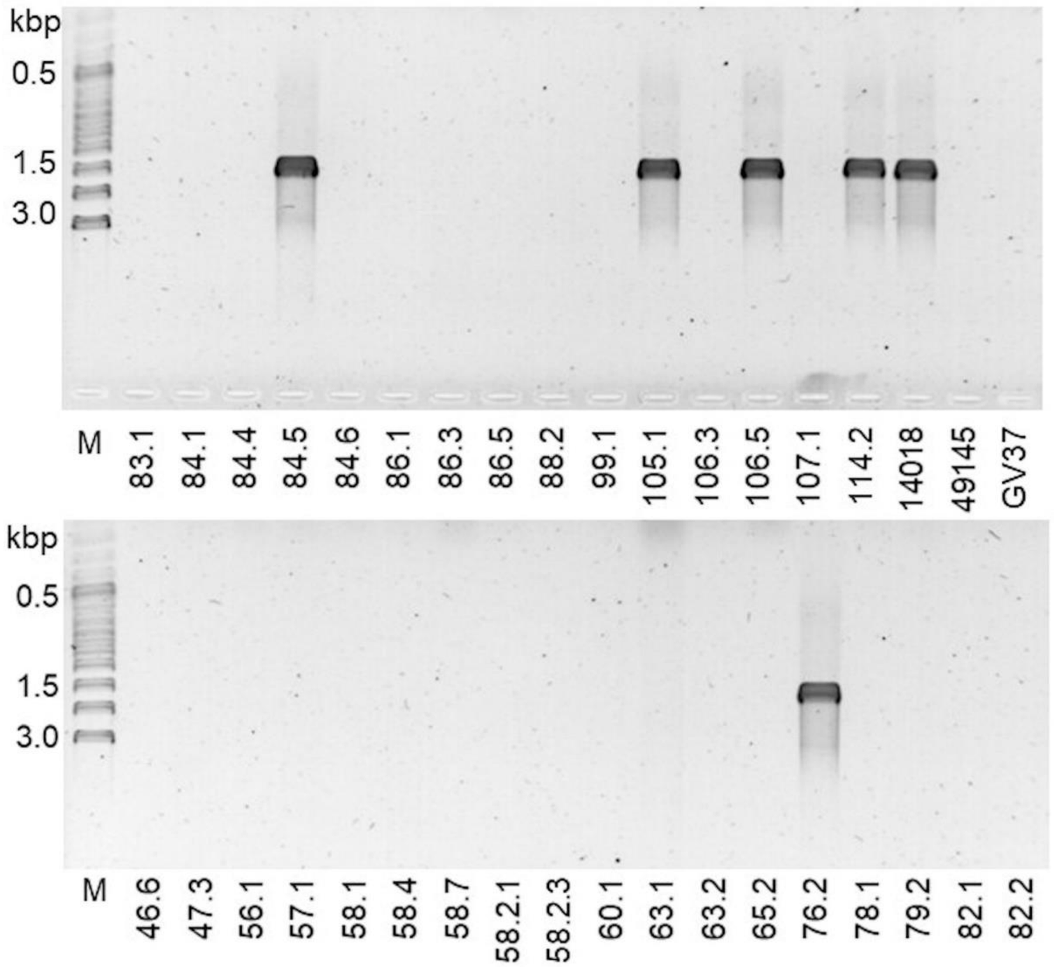

Supplementary Figure 2. Distribution of the *cna* gene across *Gardnerella* isolates. PCR analysis was conducted to detect the presence of the *cna* gene using the primers Cna-For and N-cna-Rev. The strains with available complete genome sequences were references, as follows: the *cna*-positive *G. vaginalis* ATCC 14018 (Bioproject PRJDB63) and the *cna*-negative strain 49145 (BioProject PRJNA437230). M: DNA size standards Gene Ruler 100 bp Plus DNA Ladder (Thermo Fisher Scientific).

```
                      10        20        30        40        50        60
                       |         |         |         |         |         |
105_1_Cna     QSSNDNAFTNHENIPTNKSSINNIDLIPAPTQHFGKPDGKLPEIRTSISYVDGDSTDNHA
106_5_Cna     QSSNDNAFTNRENIPTNKSSINNIDLIPAPTQHFGKPDGKLPEVRTSISYVDGDSTDNHA
114_2_Cna     QSSNDNAFTNRENIPTNKSSINNIDLIPAPTQHFGKPDGKLPEVRTSISYVDGDSTDNHA
14018_Cna     QSSNDNAFTNRENIPTNKSSINNIDLIPAPTQHFGKPDGKLPEVRTSISYVDGDSTDNHA
              **********:********************************:****************
Prim.cons.    QSSNDNAFTNRENIPTNKSSINNIDLIPAPTQHFGKPDGKLPEVRTSISYVDGDSTDNHA

                      70        80        90       100       110       120
                       |         |         |         |         |         |
105_1_Cna     NTNKTNKILRPGSKLTLRATAKFIVPEAGDVFRPYLRLAIPKILTVNANDFVVDAGSVAN
106_5_Cna     DTNKTNKILRPGSKLTLRATAKFIVPEAGDVFRPYLRLAIPKILTVNANDFVVDAGSVAN
114_2_Cna     DTNKTNKILRPGSKLTLRATAKFIVPEAGDVFRPYLRLAIPKILTVNANDFVVDAGSVAN
14018_Cna     DTNKTNKILRPGSKLTLRATAKFIVPEAGDVFRPYLRLAIPKILTVNANDFVVDAGSVAN
              :***********************************************************
Prim.cons.    DTNKTNKILRPGSKLTLRATAKFIVPEAGDVFRPYLRLAIPKILTVNANDFVVDAGSVAN

                     130       140       150       160       170       180
                       |         |         |         |         |         |
105_1_Cna     KVVQISDSKSFGNHNLGNYNLFDVYFNYNEWSSDFANEMPYLAAIKGVNHSGNVLSNDDV
106_5_Cna     KVVQISDSKSFDNHNLGNYNLFDVYFNYNEWSSDFANEMPYLAAIKGVNHSGNVLSNGDV
114_2_Cna     KVVQISDSKSFDNHNLGNYNLFDVYFNYNEWSSDFANEMPYLAAIKGVNHSGNVLSNGDV
14018_Cna     KVVQISDSKSFDNHNLGNYNLFDVYFNYNEWSSDFANEMPYLAAIKGVNHSGNVLSNGDV
              ***********.*********************************************.**
Prim.cons.    KVVQISDSKSFDNHNLGNYNLFDVYFNYNEWSSDFANEMPYLAAIKGVNHSGNVLSNGDV

                     190       200       210       220       230       240
                       |         |         |         |         |         |
105_1_Cna     IKNNQTVSIEIHTVVNSNAKTSDLVDLKNYKKGVKPLVQGRVSYMPFPTPMEYTSTIGGL
106_5_Cna     IKNNQTVSIEIHTVVNSNAKTSDLVDLKNYKKGVKPLIQGRVSYMPFPTPMEYTSTIGGL
114_2_Cna     VKNNQTVSIEIPTVVDSNANTSDLVDLQNYKKGVKPLIQGRVSYMPFPTPIEYTSTIGGL
14018_Cna     VKNNQTVSIEIPTVVDSNANTSDLVDLQNYKKGVKPLIQGRVSYMPFPTPIEYTSTIGGL
              :********** ***:***:*******:*********:************:*********
Prim.cons.    2KNNQTVSIEI2TVV2SNA2TSDLVDL2NYKKGVKPLIQGRVSYMPFPTP2EYTSTIGGL

                     250       260       270       280       290       300
                       |         |         |         |         |         |
105_1_Cna     RDFPNDRILQKNPCLVRSDLVTGWDGRDNYGFWFTGLSMETSGVDYDGRSTMVESQNGVW
106_5_Cna     RXFPXDRILQKNPCLVRSDLVTGWDGRDNYGFWFTGLSMETSGVDYDSRSTMVESQNGVW
114_2_Cna     KDFPNDKILQDNPCLVRSDLVTGWDGRDDYGFWFTGLSMETSGVDYDSRSTMVESQNGVW
14018_Cna     KDFPNDKILQDNPCLVRSDLVTGWDGRDDYGFWFTGLSMETSGVDYDSRSTMVESQNGVW
              : ** *:***.*****************:******************.************
Prim.cons.    2DFPND2ILQ2NPCLVRSDLVTGWDGRD2YGFWFTGLSMETSGVDYDSRSTMVESQNGVW

                     310       320       330       340       350       360
                       |         |         |         |         |         |
105_1_Cna     LPQLIPANIEHNQFVTIYRNWNDNDPIKLRFKIPFGSKNYALNGTKQVFKNHHLTVFRSP
106_5_Cna     LPQLIPANIEHNQFVTIYRNGNDNNPIKLRFKIPFGSKDYTLNGTKQALKNHHLTVFRSP
114_2_Cna     LPQLIPANIEHNQFVTIYRNGNDNNPIKLRFKIPFGSKDYTLNGTKQALKNHHLTVFRSP
14018_Cna     LPQLIPANIEHNQFVTIYRNGNDNNPIKLRFKIPFGSKDYTLNGTKQALKNHHLTVFRSP
              ******************** ***:*************:*:******.:***********
Prim.cons.    LPQLIPANIEHNQFVTIYRNGNDNNPIKLRFKIPFGSKDYTLNGTKQALKNHHLTVFRSP

                     370       380       390       400       410       420
                       |         |         |         |         |         |
105_1_Cna     NNNDEARKIMANIGIEKVAYGDTEVFGDILDSKYFDGWGTVPKQSRPSELLISGEFNPDS
106_5_Cna     NNNAEARKIMGNIGIEKVAYENTEVFGDILDSKYFDGWGTVPEQSRPSELLISGEFNPDS
114_2_Cna     NNNAEARKIMGNIGIEKVAYENTEVFGDILDSKYFDGWGTVPEQSRPSELLISGEFNPDS
14018_Cna     NNNAEARKIMGNIGIEKVAYENTEVFGDILDSKYFDGWGTVPEQSRPSELLISGEFNPDS
              *** ******.********* :********************:*****************
Prim.cons.    NNNAEARKIMGNIGIEKVAYENTEVFGDILDSKYFDGWGTVPEQSRPSELLISGEFNPDS
```

```
                         430       440       450       460       470       480
                           |         |         |         |         |         |
105_1_Cna         TVFGPDNISNGIWMSGKASAYIKSYYRALDCNDNYPDNTLKYPPIINKIQETVATYARSN
106_5_Cna         TVFGPDNISNGIWMSGKASASIKSYYRALDCNDNYPDSTLKYPPIINKIQETVATYARSN
114_2_Cna         TVFGPDNISDGIWMSGKASASIKSYYRALDCNDNYPDNTLKYPPIINKIQETVATYARSN
14018_Cna         TVFGPDNISNGIWMSGKASASIKSYYRALDCNDNYPDSTLKYPPIINKIQETVATYARSN
                  *********:********** ****************.**********************
Prim.cons.        TVFGPDNISNGIWMSGKASASIKSYYRALDCNDNYPD2TLKYPPIINKIQETVATYARSN

                         490       500       510       520       530       540
                           |         |         |         |         |         |
105_1_Cna         RSKVSTVPIEIVIPASLSGRVWFDANHNGIQDKGESSIIGAKVQLVKQYGDSTVIDINGN
106_5_Cna         RSKVSTVPIEIVIPASLSGRVWFDANHNGIQDKGESSIIGAKVQLVKQYGDSTVIDINGN
114_2_Cna         RSKVSTVPIEIVIPSSLSGRVWFDANHNGIQDKGESSIIGAKVQLVKQYGDSTVIDINGN
14018_Cna         RSKVSTVPIEIVIPASLSGRVWFDANHNGIQDKGESSIIGAKVQLVKQYGDSTVIDINGN
                  **************:*********************************************
Prim.cons.        RSKVSTVPIEIVIPASLSGRVWFDANHNGIQDKGESSIIGAKVQLVKQYGDSTVIDINGN

                         550       560       570       580       590       600
                           |         |         |         |         |         |
105_1_Cna         KVNPITTTSPDGSYEFTNLLPGNYVVKFTLPEGSEYFGFTYTRKGSDSSVDSDAIPGEPV
106_5_Cna         EVKPITTTSPDGYYEFTNLLPGSYVVKFTLPEGSEYFGFTYTHKGSDSSVDSDAIPGEPV
114_2_Cna         EVKPITTTSPDGYYEFTNLLPGSYVVKFTLPEGSEYFGFTYTHKGSDSSVDSDAIPGEPV
14018_Cna         EVKPITTTSPDGYYEFTNLLPGSYVVKFTLPEGSEYFGFTYTHKGSDSSVDSDAIPGEPV
                  :*:********* *********.*******************:*****************
Prim.cons.        EVKPITTTSPDGYYEFTNLLPGSYVVKFTLPEGSEYFGFTYTHKGSDSSVDSDAIPGEPV

                         610       620       630       640       650       660
                           |         |         |         |         |         |
105_1_Cna         TDRSVTEKGSAETDNSASKDSYVGNISLSAGEDKKHIDAGAFKKDEPK------------
106_5_Cna         TDRSVIENVSSETDNSTSKNSYVGNISLSAGEDKKHIDAGAFKKDKPKDPKP--------
114_2_Cna         TDRSVIENVSSETDNSTSKNSYVGNISLSAGEDKKHIDAGAFKKDEPKDPKPNTEPKNPQ
14018_Cna         TDRSVIENVSSETDNSTSKNSYVGNISLSAGEDKKHIDAGAFKKDEPKDPKPNTEPKNPQ
                  ***** *: *:*****:**:*************************:**
Prim.cons.        TDRSVIENVSSETDNSTSKNSYVGNISLSAGEDKKHIDAGAFKKDEPKDPKPNTEPKNPQ

                         670       680       690       700       710       720
                           |         |         |         |         |         |
105_1_Cna         ------------------------------------------------------------
106_5_Cna         ------------NPEPK-------------------------------------------
114_2_Cna         PKHEDPKDPKQNPNPKDPKKDKPKSKDPKPKDPNPEKDKPKDPDNKPKQNPEPKKTDPKD
14018_Cna         PKHEDPKDPKQNPNPKDPKKDKPKSKDPKPKDPNPEKDKPKDPDNKPKQNPEPKKTDPKD

Prim.cons.        PKHEDPKDPKQNPNPKDPKKDKPKSKDPKPKDPNPEKDKPKDPDNKPKQNPEPKKTDPKD

                         730       740       750       760       770       780
                           |         |         |         |         |         |
105_1_Cna         ---------------KDEPKKND----------------PKDPDPNDEPKDPKDPKPNSE
106_5_Cna         ----------SPDPKKDEPNPKDKPNNPNYQDRDVHDPKPDDPKKPDNPKKPKDDKPDN-
114_2_Cna         YNPVKPDTPKSPDPKKDDPKPKDKPNNPNYQDRDVPDTKPEDPKKPDNPTKPKDDKSDT-
14018_Cna         YNPVKPDTPKSPDPKKDDPKPKDKPNNPNYQDRDVPDTKPEDPKKPDNPTKPKDDKSDT-
                                 **:*: :*                *.**.  *:*..*** *.:.
Prim.cons.        YNPVKPDTPKSPDPKKD2PKPKDKPNNPNYQDRDVPDTKPEDPKKPDNP2KPKDDK2DTE


                         790       800       810       820       830       840
                           |         |         |         |         |         |
105_1_Cna         PKKNDPK---DPDPNDEPKKDETEDPDPKDTNPKSENPKDNENPKNPESKPDDPKSDTPK
106_5_Cna         PKEPESK---DPKQDPDTKKDDPEDPDPKDTNPKSDTPKDNENPKEPESEPDDPKSDTPD
114_2_Cna         PKEPESKEPKDPKPNDEPKKDEPEDPDPKDTNPKSENPKDNENPKNPESEPDDPKSDTPK
14018_Cna         PKEPESKEPKDPKPNDEPKKDEPEDPDPKDTNPKSENPKDNENPKNPESKPDDPKSDTPK
                  **: :.*   **. : :.***:.************:.********:***:*********.
Prim.cons.        PKEPESKEPKDPKPNDEPKKDEPEDPDPKDTNPKSENPKDNENPKNPES2PDDPKSDTPK
```

Supplementary Material

```
                    850       860       870       880       890       900
                      |         |         |         |         |         |
105_1_Cna    DPDTDDTESNDPPQYEDPQPKEDSSEPNHSEIRESDNPISSSNNDLDSNNSQSNESSFDN
106_5_Cna    GPDTDYTESNDPPQYEDPQPKEDSSEPNQSEIRELDNPIPNINNDLDSNNSQSNESSFDN
114_2_Cna    DPDTDDTESNDPPQYEDSQPKEDSSGPNHSEIRELDNPIPSSNNDLDSNNSQSNESSFDN
14018_Cna    DPDTDDTESNDPPQYEDSQPKEDSSGPNHSEIRESDNPIPNINNDLDSNNSQSNESSFDN
             .**** ***********.******* **:***** ****.. ******************
Prim.cons.   DPDTDDTESNDPPQYED2QPKEDSS2PNHSEIRE2DNPIP22NNDLDSNNSQSNESSFDN

                    910       920       930       940       950
                      |         |         |         |         |
105_1_Cna    NNADSKQERVRDKLVNTGVSTVFTAVASVAMLSLGATNKIKRYFCKGKHRQ
106_5_Cna    NNADFKQEHVRDKLVNTGVSTVFTAVASVAMLALGATNKIKSYFCKGKHRQ
114_2_Cna    NNADFKQEHVRDKLVNTGVSTVFTAVASVAMLALGATNKIKRYFCKGKHRQ
14018_Cna    NNADFKQEHVRDKLVNTGVSTVFTAVASVAMLALGATNKIKSYFCKGKHRQ
             **** ***:***********************:******** *********
Prim.cons.   NNADFKQEHVRDKLVNTGVSTVFTAVASVAMLALGATNKIK2YFCKGKHRQ
```

Supplementary Figure 3. Multiple alignment of the translated *cna* sequences obtained from *G. vaginalis* 105.1, 106.5, 114.2 (GenBank acc. nos PP874905, PP874906, PP874907) and ATCC 14018 strains (Bioproject PRJDB63). The ClustalW tool (https://npsa-pbil.ibcp.fr/) was used for multiple sequence alignment. The sequences do not include the first 26 amino acids, which are predicted to be a signal peptide. The first and last amino acids of Grd rCna are highlighted in the yellow background.

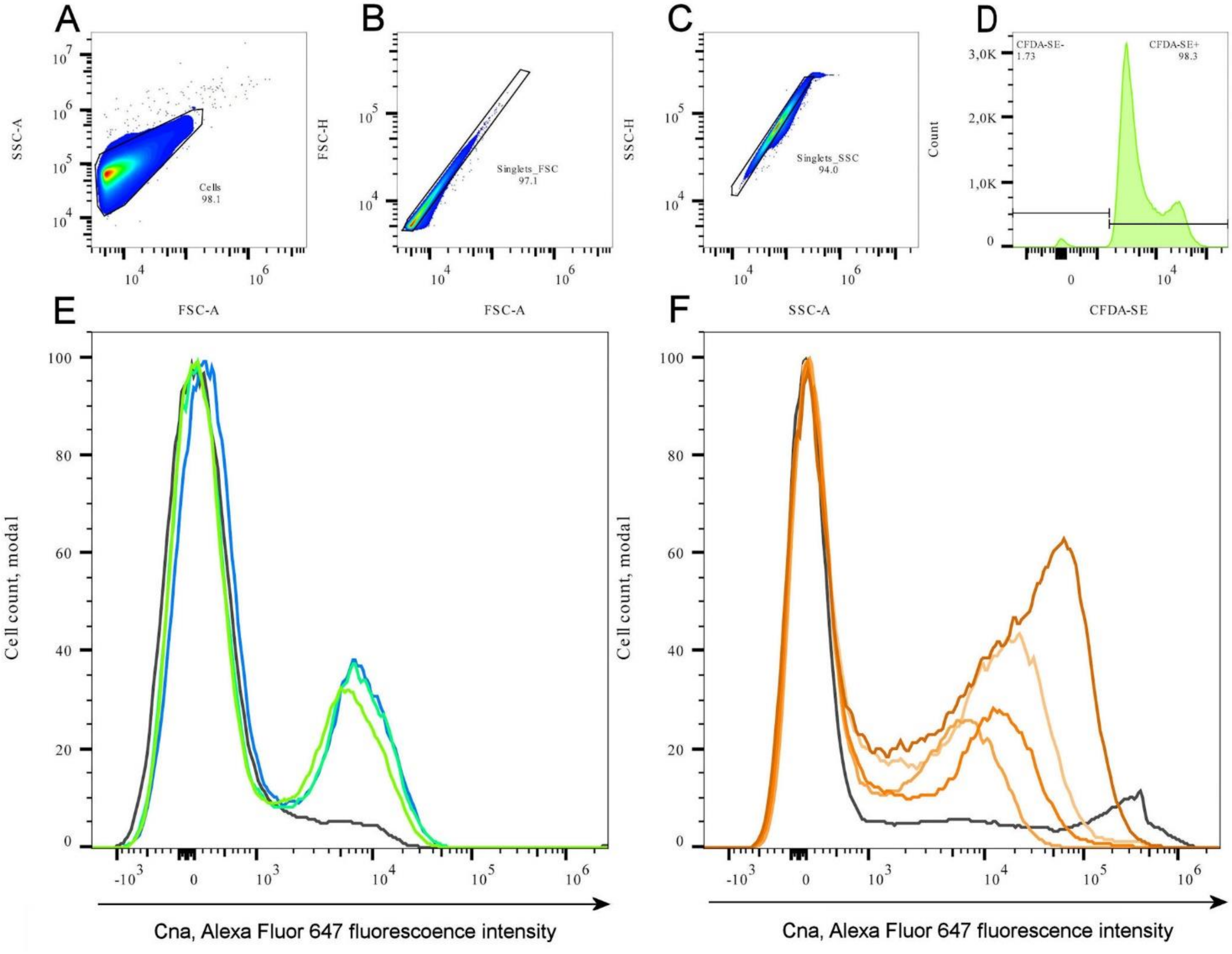

SpecimenID	Subset Name	Count	Freq. of Parent
anti-CNA MAb B4	CFDA-SE+	103095	29.9
anti-CNA MAb C1	CFDA-SE+	102242	31.6
anti-CNA MAb E2	CFDA-SE+	88225	30.8
Istype control	CFDA-SE+	89548	8.72

SpecimenID	Subset Name	Count	Freq. of Parent
anti-CNA PAbs No1	CFDA-SE+	83486	60.5
anti-CNA PAbs No2	CFDA-SE+	94452	36.6
anti-CNA PAbs No3	CFDA-SE+	103334	33.7
anti-CNA PAbs No4	CFDA-SE+	84497	48.4
PAbs NEG control	CFDA-SE+	98557	25.6

Supplementary Figure 4. Gating strategy and flow cytometry measurements of *G. vaginalis* ATCC 14018 cells stained with CFDA-SE and exposed to antibodies targeting the Grd rCna protein. (A) Cells were gated using FSC-A vs. SSC-A to select the population of interest. (B, C) Singlet cells were identified through FSC-A vs. FSC-H and SSC-A vs. SSC-H gating. (D) CFDA-SE-positive cells were determined using bisector gates established from a negative control, with 98.3% classified as CFDA-SE-positive. (E, F) Alexa Fluor 647 fluorescence signal intensities were measured for CFDA-SE-positive cells probed with anti-rCna MAbs B4, C1, and E2 (E) or four anti-rCna PAbs (F). Isotype control antibodies and irrelevant PAbs were used as controls to account for nonspecific binding.

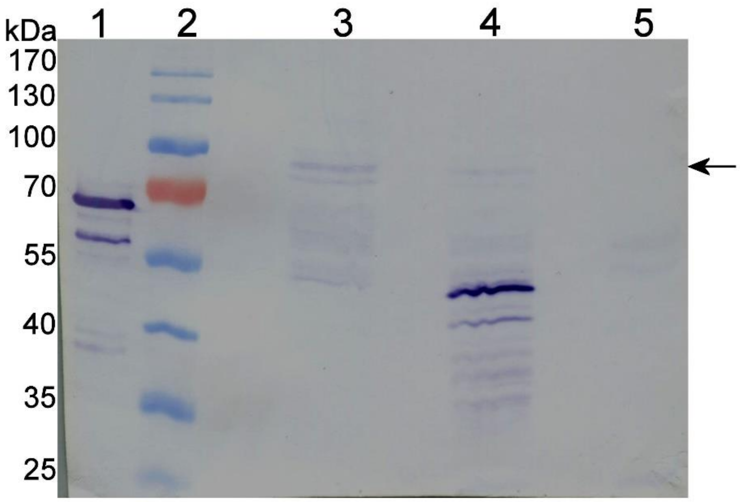

Supplementary Figure 5. Western blot analysis of *G. vaginalis* cell wall extracts using anti-rCna MAb B4. Lane 1, 0.2 µg of the purified rCna protein; lane 2, PageRuler™ Prestained Protein Ladder (Thermo Fisher Scientific), the numbers on the left side indicate mol. weight of standards. Cell wall extracts containing approx. 0.2 mg of total protein from *G.vaginalis* strains 114.2 (lane 3), ATCC 14018 (lane 4), and ATCC 49145 (lane 5). The arrow on the right side indicates the position corresponding to the Grd Cna protein band.

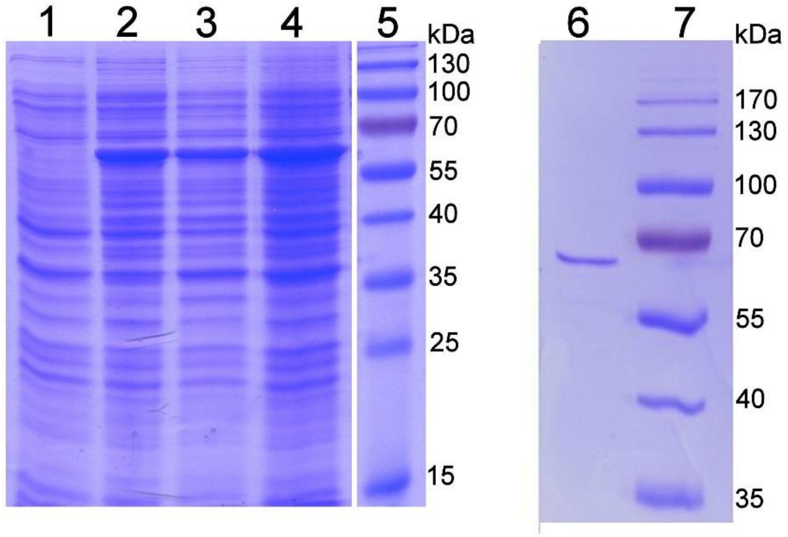

Supplementary Figure 6. SDS-PAGE analysis of Grd rCna in *E. coli* lysates and the purified rCna protein. Soluble (lane 2) and insoluble (lane 3) fractions of cell lysate, and total cell lysate (lane 4) containing rCna. Lane1, total cell lysate before adding IPTG (control). Lane 6, 0.2 µg of purified rCna protein. Lane 5, 7, protein molecular weight standards PageRuler™ Prestained Protein Ladder (Thermo Fisher Scientific).
